# Menstrual bleeding patterns: A community-based cross-sectional study among women aged 18-45 years in Southern Brazil

**DOI:** 10.1186/1472-6874-11-26

**Published:** 2011-06-07

**Authors:** Iná S Santos, Gicele C Minten, Neiva CJ Valle, Giovana C Tuerlinckx, Alessandra B Silva, Guilherme AR Pereira, Joaquim F Carriconde

**Affiliations:** 1 1Postgraduate Program in Epidemiology, Federal University of Pelotas, Pelotas, CP464, RS, Brazil

## Abstract

**Background:**

Population variation in the duration and amount of menstrual bleeding has received little attention in the literature. This study describes these characteristics and investigates the distribution of self-perceived amount of menstrual bleeding according to socio-demographic, behavioral, and reproductive characteristics.

**Methods:**

A community-based cross-sectional study was conducted among 18-45 years old women users of the 31 primary health care (PHC) facilities in Pelotas city (Brazil). Interviews with structured questionnaire were carried out in the waiting rooms during two work shifts. Heaviness of menstrual bleeding was determined through the answer to the question: "Usually how much blood do you lose in every period?" Crude and adjusted analyses through Poisson regression took into account the aggregation per PHC facility.

**Results:**

A total of 865 women were enrolled. Prevalence of heavy menstrual flow was 35.3% (95% CI 32.1-38.6%). In adjusted analyses, heavy menstrual bleeding was higher among the older, less educated and obese women, with higher number of pregnancies and who reported longer menstrual periods, extra-menstrual bleeding and clots in the flow. Use of hormonal contraceptive methods was protective against heavy menses.

**Conclusion:**

Heavy menstrual bleeding is highly prevalent at the community level, and is associated with socio-demographic and anthropometric women's characteristics, as well as with duration of menstruation, extra-bleeding and presence of clots.

## Background

Menstrual blood loss can vary greatly in quantity and identification of heavy blood loss which may have important impact over women wellbeing and health, may be particularly difficult [1]. Judging heaviness of loss is a complex decision based on personal norm (past experience of periods), difficulty in containing the volume of loss (sanitary protection used), and pattern of loss (presence of clots and flooding) [2].

Although abnormal bleeding is among the major causes of gynecologic morbidity [3,4] and in some settings a leading indicator for hysterectomy [5,6], population variation in the duration and amount of menstrual bleeding has been the subject of few researches worldwide. This study describes the duration and amount of menstrual bleeding and investigates the association between heavy menstrual flow and socio-demographic, behavioral, and reproductive characteristics in a community-based sample of women aged 18-45 years.

## Methods

This was a community-based cross-sectional study carried out in all the 31 Primary Health Care (PHC) facilities within the Unified Health System (Sistema Unico de Saude - SUS) in the urban zone of Pelotas city, in Brazil, between August 2006 and February 2007. The study included women aged 18 to 45 years who were in the waiting rooms for consultations for any reason, and also the accompanying persons, provided that they too were users of the PHC facilities of the city.

Four interviewers received prior training to apply a standardized pre-coded questionnaire and to take anthropometric measurements. The procedures were implemented during two work shifts of each PHC unit (two mornings of two consecutive days). To those who attended eligibility criteria and agreed participate a structured questionnaire on demographic (age and skin color), socio-economic (family monthly income, marital status and schooling) and behavioral characteristics (smoking, weekly consumption of alcoholic beverages and daily consumption of caffeine from coffee and maté drink) was applied. Current or previous medical diagnosis of anemia was explored.

Information regarding reproductive characteristics (current pregnancy, number of pregnancies and number of abortions), menstrual features (age at menarche, duration in days and intensity of usual menstrual periods, extra-menstrual bleeding, and presence of clots), and contraceptive method in use was gathered. Heaviness of menstrual bleeding was determined through the answer to the question: "Usually how much blood do you lose in every period? The intensity of menses was then classified as heavy, light or normal, respectively for those who replied "great quantity", "small quantity" or "normal". For those currently pregnant or at menopause, questions on menstrual characteristics were referred to their last periods. As a way of ascertaining consistency of the reported intensity of blood loss, women were asked to describe the type, size and number of sanitary pads used during the day of heaviest bleeding of their current typical periods. At the laboratory, sanitary pads of the same trade mark and size were soaked with water, then the excess water was squeezed and sanitary pads were weighted in a 100 g precision scale. This value was multiplied by the number of sanitary pads used in the day of heaviest bleeding of a typical period.

An aluminum anthropometer of 1 cm accuracy and a UNICEF dry balance of accuracy 100 g and capacity 150 kg were used for anthropometric measurements. The body mass index (BMI) was calculated as the weight (kg) divided by the squared height (in meters). Quality control to check reliability of some of the questions was done by means of applying a reduced questionnaire by telephone to approximately 10% of the interviewees randomly selected. The *kappa *statistic of agreement with a reported previous medical diagnosis of anemia was 0.80.

Intensity of menstrual flow was stratified according to socio-demographic and behavioral characteristics, as well as by type of contraceptive method and by other features of the menstruation. The strength of the association between independent variables and heavy blood flow was assessed through Poisson regression with robust variance. Prevalence ratios (PR) and 95% confidence intervals (95% CI) were calculated. For multivariable analysis purposes, a hierarchical model of determination, based on a conceptual framework, was constructed. This model allows quantifying the contribution of each level to heavy blood loss. In the higher level there were socio-demographic characteristics (age, skin color, marital status, income, and education); in the second level there were behavioral characteristics (smoking, alcohol and caffeine consumption) and BMI; in the third level, reproductive variables (age at menarche, number of pregnancies, number of abortions, and contraceptive method) and history of anemia; in the fourth most proximal level, there were other characteristics of the menstrual flow (duration, extra-bleeding and clots). Association between all independent variables was tested and two interaction terms age*education and number of pregnancies*contraceptive method were entered in the adjusted model at the first and third level, respectively. Confounder control was carried out for variables in the same level and for variables in superior levels. As contraception may be used to treat menstrual bleeding the variable "contraceptive method in use" was grouped in four categories before adjusted analyses: pill or injectables; tubal ligation; intra-uterine device (IUD); and all other methods (including male preservative) or none. Analyses were performed with the Stata 9.0 software and took into account the clustering of the data in PHC facilities.

The study protocol was approved by the Research Ethics Committee of the School of Medicine of the Federal University of Pelotas and by the Municipal Department of Health and Social Wellbeing of Pelotas. Before enrollment, the participants signed an informed consent.

## Results

Since parameters for sample size calculation were set to attend the aims of another research question to which the current study was linked [7], power of the study was calculated post hoc. The resulting sample of 865 women has a 85% power to detect relative risks ≥ 1.4 significant at alpha of 0.05, for exposures with a prevalence of 30% or greater and an expected frequency of disease in unexposed group of ≥ 30%.

**Figure 1:**
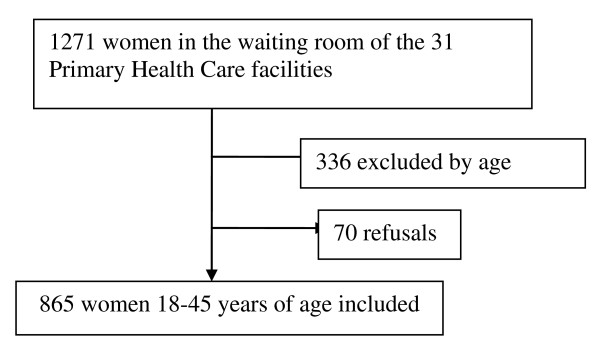
**Flow chart showing number of women in the waiting rooms and women selected to the study**.

Figure 1 contains a flow chart describing the number of women approached and selected. Non-responders (7.5%) were similar to women in the sample in terms of average age (32 ± 8 years). A total of 865 women 18-45 years of age were enrolled in the study. Prevalence of reported heavy, normal and light menstrual flow was 35.3% (95% CI 32.1-38.6%), 39.4% (36.2-42.8%), and 25.3% (22.4-28.3%), respectively. Several types of absorbent pads were worn during menstruation, the disposable sanitary pads being the most employed (reported by 99.5% of the women). After squeezed, thin, regular, maxi and nocturnal size sanitary pads weighted in average five times more than when dried: 30, 36, 40, and 54 grams, respectively. A disposable sanitary napkin weighted in average 139 grams (equivalent to about 4 regular sanitary pads), whereas a washable sanitary napkin and a tampon weighted in average 80 grams (about 2 regular sanitary pads each). Average weight of sanitary pads used during the heaviest day of the menstrual period by women who reported heavy, normal and light bleeding was 152.7 g (95%CI 145.1-160.4), 120.4 g (95%CI 115.4-125.5) and 102.6 g (95%CI 96.4-108.8), respectively (equivalent to 4.2, 3.3 and 2.8 regular size sanitary pads per day).

Table 1 shows the sample distribution and reported intensity of menstrual flow according to socio-demographic and behavioral variables. Intensity of bleeding was associated with woman age, level of education and BMI. Higher age, lower level of education and obesity were characteristics associated with higher probability of reporting heavy than light or normal bleeding in the menses.

**Table 1:** Sample distribution and reported intensity of menstruation according to socio-demographic and behavioral characteristics

		Reported intensity menstrual flow			
		
Variable	N (%)	Heavy	Normal	Light	p
		**304 (35.3)**	**340 (39.4)**	**218 (25.3)**	
Age (years)					<0.001
18-24	204 (23.6)	68 (33.3)	100 (49.0)	36 (17.6)	
25-29	159 (18.4)	36 (22.8)	70 (44.3)	52 (32.9)	
30-34	171 (19.8)	57 (33.3)	67 (39.2)	47 (27.5)	
35-39	149 (17.2)	61 (41.2)	46 (31.1)	41 (27.7)	
40-45	182 (21.0)	82 (45.3)	57 (31.5)	42 (23.2)	
Skin color					0.2
White	641 (74.1)	218 (34.1)	250 (39.1)	171 (26.8)	
Black/mixed	224 (25.9)	86 (38.6)	90 (40.4)	47 (21.1)	
Marital status					0.2
Married/with a partner	625 (72.3)	218 (35.0)	240 (38.5)	165 (26.5)	
Single	155 (17.9)	48 (31.0)	70 (45.2)	37 (23.9)	
Divorced, widowed	84 (9.7)	38 (45.2)	30 (35.7)	16 (19.0)	
Income (MW)^1^					0.5
≤ 1	181 (21.0)	69 (38.3)	66 (36.7)	45 (25.0)	
1.01-2.0	355 (41.1)	130 (36.7)	145 (41.0)	79 (22.3)	
2.01-3.0	168 (19.5)	55 (32.7)	63 (37.5)	50 (29.8)	
> 3	159 (18.4)	50 (31.4)	65 (40.9)	44 (27.7)	
Education level (years)					0.001
0-4	179 (20.7)	72 (40.2)	60 (33.5)	47 (26.3)	
5-8	394 (45.6)	153 (38.9)	137 (34.9)	103 (26.2)	
≥9	291 (33.7)	79 (27.2)	143 (49.3)	68 (23.4)	
Weekly alcohol consumption					0.1
No	773 (89.5)	277 (35.9)	307 (39.8)	187 (24.3)	
Yes	91 (10.5)	27 (29.7)	33 (36.3)	31 (34.1)	
Smoking					0.1
No	409 (47.3)	134 (32.8)	180 (44.1)	94 (23.0)	
Former smoker	172 (19.9)	63 (36.6)	59 (34.3)	50 (29.1)	
Yes	283 (32.8)	107 (37.9)	101 (35.8)	74 (26.2)	
Caffeine consumption (mg/day)					0.7
0-100	201 (23.3)	68 (34.0)	81 (40.5)	51 (25.5)	
100.1-200.0	272 (31.5)	88 (32.4)	107 (39.3)	77 (28.3)	
200.1-300.0	163 (18.9)	59 (36.4)	65 (40.1)	38 (23.5)	
> 300	228 (26.4)	89 (39.0)	87 (38.2)	52 (22.8)	
History of anemia					0.05
No	265 (30.7)	86 (32.6)	97 (36.7)	81 (30.7)	
Yes	599 (69.3)	218 (36.5)	234 (40.3)	137 (22.9)	
BMI^2^					0.008
≤ 24.9	461 (53.7)	156 (33.9)	192 (41.7)	112 (24.3)	
25.0-29.9	237 (27.6)	74 (31.2)	102 (43.0)	61 (25.7)	
≥ 30.0	161 (18.7)	73 (45.6)	44 (27.5)	43 (26.9)	

^1^MW = Minimum Wages; ^2^BMI: Body Mass Index;

There were 35 women currently pregnant in the sample and 19 that were at the menopause (15 of them had a surgical menopause due to hysterectomy). Among the remaining the contraceptive method more frequently used was the pill (51%), followed by male barrier contraception (15%) and tubal ligation (11.5%). Higher number of pregnancies, tubal ligation and use of IUD were associated with increased prevalence of heavy menstrual flow. Women reporting no use of any contraceptive method also had higher prevalence of heavy than light or normal bleeding flow.

Average duration of menstrual period was 4.4 days (95%CI 4.2-4.5 days) (ranging from 1 to 30 days) with most of them (61.2%) replying 1, 2, 3 or 4 days and more than one third (39%) reporting menstrual periods lasting five days or more. The number (and percentage) of women reporting menses lasting 6, 7, 8, 9, and 10 days was 30 (3.5%), 94 (10.9%), 8 (0.9%), 2 (0.2%), and 4 (0.5%), respectively. Most of the women with menses lasting 6-10 days (72.3%) reported heavy menstrual flow. Extra-menstrual bleeding was reported by 9% of the women and presence of clots by 54%. Those with longer duration of menstrual periods (≥ 5 days) and who reported extra-menstrual bleeding or presence of clots in the menstrual blood presented higher prevalence of heavy flow (Table 2).

**Table 2:** Sample distribution and reported intensity of menstruation according to reproductive and menstrual characteristics

		Reported intensity menstrual flow			
		
Characteristic	N (%)	Heavy	Normal	Light	p
		304 (35.3)	340 (39.4)	218 (25.3)	
Age at menarche (years)					0.8
≤ 10	73 (8.5)	32 (43.8)	26 (35.6)	15 (20.5)	
11-12	363 (42.2)	128 (35.4)	143 (39.5)	91 (25.1)	
13-14	330 (38.3)	109 (33.1)	134 (40.7)	86 (26.1)	
≥ 15	95 (11.0)	32 (33.7)	37 (38.9)	26 (27.4)	
Number of pregnancies					<0.001
0	96 (11.1)	23 (24.0)	46 (47.9)	27 (28.1)	
1-3	603 (69.8)	197 (32.7)	251 (41.6)	155 (25.7)	
≥ 4	165 (19.1)	84 (51.5)	43 (26.4)	36 (22.1)	
Number of abortions					0.3
0	588 (76.6)	215 (36.6)	233 (39.7)	139 (23.7)	
≥1	180 (23.4)	66 (36.9)	61 (34.1)	52 (29.1)	
Contraceptive method currently in use^1^					<0.001
None	26 (3.2)	14 (53.9)	6 (23.1)	6 (23.1)	
Pill	416 (51.5)	102 (24.5)	177 (42.6)	137 (32.9)	
IUD	44 (5.5)	25 (56.8)	10 (22.7)	9 (20.5)	
Tubal ligation	93 (11.5)	63 (68.5)	16 (17.4)	13 (14.1)	
Injectable hormones	35 (4.3)	8 (22.9)	17 (48.6)	10 (28.6)	
Male preservative	121 (15.0)	46 (38.3)	52 (43.3)	22 (18.3)	
Others	73 (9.0)	23 (31.5)	40 (54.8)	10 (13.7)	
Duration of menstrual periods (days)					<0.001
≤ 3	332 (38.4)	67 (20.2)	124 (37.5)	140 (42.3)	
4	197 (22.8)	50 (25.4)	104 (52.8)	43 (21.8)	
≥ 5	335 (38.8)	187 (56.0)	112 (33.5)	35 (10.5)	
Extra-menstrual bleeding					0.001
No	788 (91.2)	262 (33.3)	320 (40.7)	204 (26.0)	
Yes	76 (8.8)	42 (55.3)	20 (26.3)	14 (18.4)	
Presence of blood clots					<0.001
No	398 (46.2)	98 (24.6)	181 (45.5)	119 (29.9)	
Yes	464 (53.8)	206 (44.4)	159 (34.3)	99 (21.3)	

^1^Currently pregnant (n = 35) and women at menopause (n = 19) excluded

Table 3 shows crude and adjusted prevalence ratios (PR) for heavy menstrual bleeding according to variables kept in the final model. Heavy menstrual bleeding was associated with age and education level. Women 40-45 years old had a probability 31% higher of presenting heavy blood loss (PR 1.31; 95% CI 1.02-1.69) when compared to 18-24 year old women taken as the reference group. On the other hand, probability of reporting heavy periods was 30% lower among more educated women in comparison to those with 0-4 years of formal education. There was no interaction between age and education over the risk of presenting heavy menstrual flow.

**Table 3:** Prevalence ratios (PR) with 95% confidence intervals (95%CI) for reported heavy menstrual bleeding

Level	Variable	Crude PR (95% CI)	p	**Adjusted PR (95% CI)**^**1**^	p
1	Age (years)		0.0006		0.0008
	18-24	1		1	
	25-29	0.68 (0.48-0.97)		0.67 (0.47-0.94)	
	30-34	1.00 (0.75-1.33)		0.98 (0.73-1.31)	
	35-39	1.24 (0.94-1.63)		1.19 (0.90-1.57)	
	40-45	1.36 (1.06-1.75)		1.31 (1.02-1.69)	
1	Education level (years)		0.001^4^		0.005
	0-4	1		1	
	5-8	0.97 (0.78-1.20)		1.01 (0.82-1.26)	
	≥9	0.67 (0.52-0.88)		0.71 (0.55-0.92)	
2	BMI^2^		0.006		0.01
	≤ 24.9	1		1	
	25.0-29.9	0.92 (0.73-1.15)		0.90 (0.72-1.13)	
	≥ 30.0	1.35 (1.09-1.66)		1.29 (1.04-1.59)	
3	Number of pregnancies		<0.001^4^		0.001^4^
	0	1		1	
	1-3	1.36 (0.94-1.99)		1.20 (0.81-1.77)	
	≥ 4	2.15 (1.46-3.17)		1.58 (1.02-2.43)	
3	Contraceptive method currently in use^3^		<0.001		<0.001
	Pill/Injectable hormones	0.64 (0.51-0.82)		0.66 (0.52-0.84)	
	IUD	1.50 (1.10-2.04)		1.36 (0.99-1.87)	
	Tubal ligation	1.81 (1.45-2.25)		1.59 (1.25-2.01)	
	Preservative/other methods/none	1		1	
4	Duration of menstrual periods (days)		<0.001^4^		<0.001^4^
	≤ 3	1		1	
	4	1.25 (0.91-1.73)		1.21 (0.88-1.67)	
	≥ 5	2.77 (2.19-3.50)		2.27 (1.80-2.87)	
4	Extra-menstrual bleeding		<0.001		0.03
	No	1		1	
	Yes	1.66 (1.33-2.07)		1.28 (1.01-1.63)	
4	Presence of blood clots		<0.001		<0.001
	No	1		1	
	Yes	1.80 (1.48-2.20)		1.44 (1.18-1.76)	

^1^Confounder control was carried out for variables in the same level and variables in higher levels; ^2^BMI: Body Mass Index; ^3^Currently pregnant (n = 35) and women at menopause (n = 19) excluded; ^4^test for linear trend

None of the behavioral characteristics were associated with heavy blood loss in multivariable analysis. After adjusting for age and education, obese women had a 29% increase in probability of reporting heavy bleeding (PR 1.29; 95% CI 1.04-1.59) when taking women with BMI ≤ 24.9 as the comparison group. With regard to the number of pregnancies, after allowing for age, education and BMI, women with ≥ 4 pregnancies presented an almost 60% increase in probability of reporting a heavy menstrual loss in comparison to never pregnant women (PR 1.58; CI95% 1.02-2.43). The use of hormonal contraception was protective against reported heavy menstruations (0.66; CI95% 0.52-0.84). There was no interaction between parity and type of contraceptive method over risk of heavy menstrual flow.

Adjusting for age, education, BMI, number of pregnancies, and contraceptive method in use, all other three characteristics of menstruation remained associated with heavy blood loss. Those with menstrual periods lasting five days or more had a 2.3 fold increase in probability of reporting heavy menses. Women who reported extra-menstrual bleeding and clots in menstrual blood presented respectively a 28% and 44% increase in probability of also reporting heavy bleeding.

Since hormonal contraceptives alter a woman's natural menstrual bleeding pattern [8], analyses on intensity of menstrual flow were repeated after excluding women who reported use of hormones as a contraceptive method (oral or injectable). Except for BMI and number of pregnancies (characteristics that were no longer statistically associated with heavy menstrual bleeding) all the other associations remained similar to what was observed when all women were included in the analyses.

## Discussion

The main new findings of this study relate to the high prevalence of heavy menstrual bleeding in this population and to the increased prevalence in women with little formal education. The observed prevalence (35%) is equal to the prevalence reported by Santer et al among women aged 25 to 44 in Scotland [9] and about four times higher than the one described in a literature review on the prevalence of self-reported heavy bleeding in developing countries (4-9% in most of the studies) [1]. It should be noted however that this last difference may be due to the fact that the presence of a symptom does not necessarily constitute morbidity as it may not be causing physical, social or emotional distress or interfering with function or lead on to any of these.

There are difficulties in comparing studies on reported vaginal bleeding for several reasons. Differences in prevalence may be due to the way the blood loss was assessed: face-to-face interviews, self-applied questionnaires, application of pictorial blood loss assessment chart or through objective measurement of menstrual blood loss (alkaline hematin method) [8]. Although more accurate, objective measurements or pictorial charts have their application limited in population surveys since the two methods rely on information collected during all days of one or more menstrual periods. Structured or semi-structured questionnaires with proven validity are largely employed in population-based epidemiological studies. However, no studies assessing validity of reported intensity of menstrual flow against a gold standard among Brazilian women were found at the literature. The question used in the current study to assess heaviness of the periods showed to be consistent with the weight of sanitary pads used by women who reported heavy menstrual flow.

Prevalence ratios for heavy flow increased with women's age. Shapley et al [10] and Janssen et al [11] in population-based cross-sectional studies conducted in England and in The Netherlands, respectively, reported increased prevalence of menorrhagia with increasing age. The association between BMI and heavy menstrual flow had also been reported among women approaching menopause (42-52 years of age) [12]. Prevalence of heavy menstrual flow was higher among parous than among nulliparous women, a finding that was also described by others [11]. In spite of having biological plausibility, this study did not find association between present or past history of anemia and higher prevalence of heavy menstrual flow. In adjusted analysis, hormonal contraception was protective against heaviness.

The reported prevalence of extra-menstrual bleeding (9%) is similar to findings from other studies conducted in developing countries (1-11% of women report spotting or inter-menstrual bleeding) [1]. Prevalence of clots (54%) detected in the current study is comparable to the one described by Parker et al [13] among Australian teenagers (58%). No studies were found reporting prevalence of heavy menstrual flow according to the occurrence of extra-menstrual bleeding or clots. Also, no studies were located in which low formal education had been investigated as an exposure variable, preventing comparison with current finding of higher prevalence of heavy periods among less educated women. In this sample woman education was associated with other factors related to increased frequency of heavy menstrual flow like age, number of pregnancies, type of contraceptive method in use, and extra-menstrual bleeding. Low educated women in general were older and presented a higher number of pregnancies than more educated women. Prevalence of extra-menstrual bleeding was higher among less educated women (14% *versus *6%; p < 0.001). Use of hormonal contraception was less prevalent among low educated women than among those with nine years or more of formal education (51% against 61%; p = 0.005).

One strength of this study was its community setting which allowed exploring patterns of menstrual bleeding among a population of women who were not selected because they were presenting a gynecological symptom. Several strategies were adopted to prevent information bias. Before starting data collection, interviewers were trained in techniques of interview and in application of the questionnaire; 10% of the interviews were repeated by the supervisor of the study to check for reliability of the answers; and the coerence of the reported intensity of the blood flow was checked.

One limitation of this sampling strategy is that it was developed among the users of PHC units and therefore the sample was not representative of the population of the city as a whole. The PHC units serve the poorest 30% of the population living in the urban zone of the city [14], which leads to a high homogeneity to the sample regarding socio-economic and behavioral characteristics. Among additional limitations of the current study some menstrual features (like regularity of the menstrual cycle, presence of symptoms during menses), reported by others as factors associated to heavy menstrual flow had not been investigated. Also, since women have not been requested to classify their menses as normal or abnormal, reported heavy menses in this study probably includes heavy normal flow and abnormally heavy bleeding. Furthermore, some error could have being introduced if women did not "fill" each pad before changing. We asked women to report size and amount of pads used during the heaviest day of the period with the intention to prevent as most as possible this source of error. Since the objective of the question was not to obtain the exact amount of blood lost but instead, to check whether there was consistency between the intensity of flow reported and the absorbent capacity of the sanitary pads used, this type of error was assumed for the entire sample. However, it is important to highlight that the weights obtained do not correspond to the exact amount of menstrual blood loss.

## Conclusions

Reported heavy menstrual bleeding is highly prevalent at the community level and is related to older age, low formal education, obesity, higher number of pregnancies, as well as with other features of menstruation, like longer periods, extra-menstrual bleeding, and clots in the flow. On the other hand, hormonal contraception is protective against heavy menses.

## Competing interests

The authors declare that they have no competing interests.

## Authors' contributions

ISS conceived of the study and participated in its design, coordination, analyses, and interpretation of the results, and drafted the manuscript. GCM and GCT participated in the design of the study, planned the logistic for data collection, and coordinated field work. NCJV performed the statistical analysis. ABS, GARP and JFC participated in the coordination of the study, edited data set and performed preliminary analyses. All authors read and approved the final manuscript.

## Pre-publication history

The pre-publication history for this paper can be accessed here:

http://www.biomedcentral.com/1472-6874/11/26/prepub
